# Naringenin cationic lipid-modified nanoparticles mitigate MASLD progression by modulating lipid homeostasis and gut microbiota

**DOI:** 10.1186/s12951-025-03228-x

**Published:** 2025-03-04

**Authors:** Lu Dong, Wenyong Lou, Congfei Xu, Juan Wang

**Affiliations:** 1 School of Food Science and Engineering, South China University of Technology, Guangzhou, Guangdong Province 510641 China; 2 School of Biomedical Sciences and Engineering, South China University of Technology, Guangzhou International Campus, Guangzhou, 511442 China

**Keywords:** Naringenin, Cationic nanoparticles, MASLD, Gut microbiota

## Abstract

Naringenin (NAR) possesses various pharmacological activities including antioxidant, anti-inflammatory, and hepatoprotective effects. However, its therapeutic efficacy is limited by its hydrophobic and crystalline nature. This study aimed to investigate the therapeutic potential and molecular mechanisms of NAR efficiently loaded into cationic nanoparticles (NP-NAR) for treating metabolic dysfunction-associated steatotic liver disease (MASLD) in a mouse model. The results demonstrated that NP-NAR effectively ameliorated lipid metabolism dysbiosis, oxidative stress, insulin resistance, and inflammation in MASLD mice. Transcriptomic analysis and molecular data revealed that NP-NAR promoted fatty acid oxidation via activation of the PPAR signaling pathway, reduced hepatic lipid uptake and lipogenesis by inhibiting the expressions of key genes including CD36, ACC, and FASN. Moreover, NP-NAR modulated cholesterol metabolism by inhibiting the classical bile acid synthesis pathway. 16 S rDNA gene sequencing revealed a disbalanced gut microbiota in MASLD mice, whereas NP-NAR treatment statistically reversed the abundance changes of several intestinal bacteria at the phylum and genus levels, which partly contributed to the balance in intestinal metabolite production, including short-chain fatty acids. In conclusion, these findings suggest that NP-NAR may be a promising candidate for the treatment of obesity-associated MASLD, offering new insight into the mechanisms underlying NAR’s efficacy against MASLD.

**Graphical Abstract:**

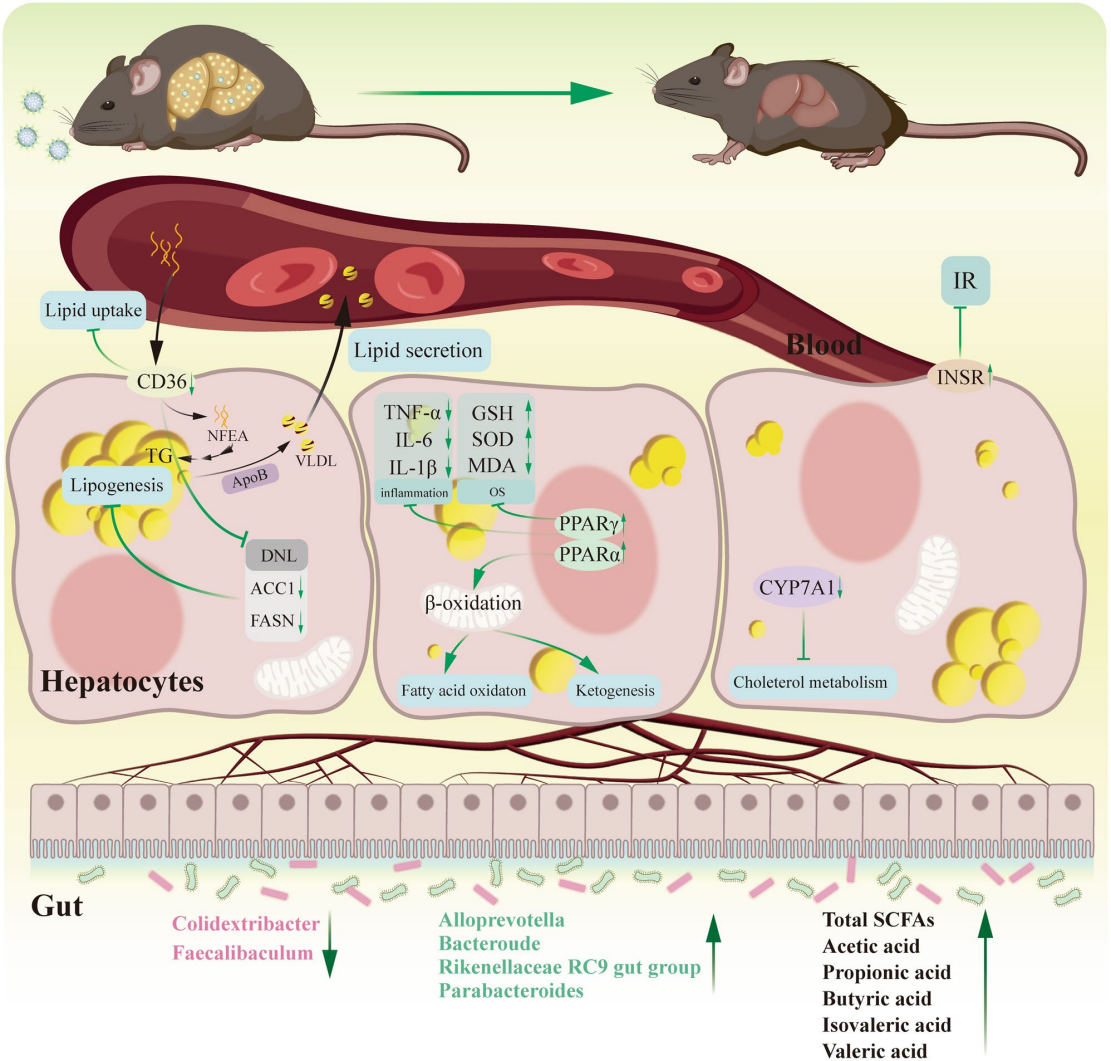

**Supplementary Information:**

The online version contains supplementary material available at 10.1186/s12951-025-03228-x.

## Background

Due to the obesity epidemic and the prevalence of “Western lifestyle”, characterized by energy-dense, nutrient-poor foods alongside sedentary, metabolic dysfunction-associated steatotic liver disease (MASLD) has become increasingly common in recent decades [1, 2]. MASLD is a multifaceted condition influenced by factors such as environment, metabolism, the microbiome, comorbidities, and genetic predisposition [3]. Currently, no approved treatments exist for MASLD, although many drugs have shown promise in clinical trials, including farnesoid X receptor (FXR) agonist (obeticholic acid), peroxisome proliferator-activated receptor (PPAR) agonists (elafibrinor), and C-C chemokine receptor (CCR) antagonist (cencriviroc) [3]. However, these drugs have some issues with low efficiency, organ toxicity, or the compensatory responses [4–6]. Concomitantly, natural products (NPs) like the naringenin (NAR) have attracted increasing attention for their pharmacological properties, including anti-inflammatory, antioxidant, hypoglycemic, hepatoprotective, and anticancer activities [7–10]. NAR, primarily found in citrus fruits like grapefruits, exists in such low quantities that obtaining it directly from fruits is impractical [11]. Furthermore, its hydrophobic and crystalline nature contributes to low oral bioavailability and instability, posing a great challenge for its application.

Cationic lipid-assisted nanoparticle (CLAN) have been utilized for delivering small molecule drugs and nucleic acid-based drugs [12–14]. Incorporating small amounts of cationic lipids into nanocarriers facilitates their adsorption onto cell surfaces with anionic characteristics, significantly enhancing the permeability and cellular uptake of nanomedicines [15–17]. Importantly, all components of CLAN have been deemed non-toxic and are either clinically validated or under clinical investigation [12].

The gut microbiota paly a crucial role in hepatic lipid metabolism through the gut-liver axis [18]. Therefore, the composition and metabolites of the gut microbiota have emerged as critical elements in regulating the pathologic process of MASLD. Normally, commensals maintain a healthy intestinal barrier through a variety of mechanisms, such as the production of short-chain fatty acids (SCFAs) [19], while a healthy liver and its immune system is able to maintain a balance between immunity and tolerance [20]. Nonetheless, a prolonged Western diet impair the gut barrier, leading to low-grade systemic inflammation and dysbiosis [21]. Repairing the intestinal barrier and restoring SCFA levels are key therapeutic goals in managing MASLD.

This study developed naringenin-loaded cationic nanoparticles (NP-NAR) using CLAN delivery system to achieve efficient transport and targeted delivery. Using an high-fat diet (HFD)-induced MASLD mouse model, the study investigated the effects of NP-NAR on MASLD progression and explored the role of the gut barrier in this process. Transcriptomic analysis was employed to uncover potential mechanisms underlying NP-NAR’s therapeutic effects. This study will provide a safe and effective delivery method for fat-soluble sensitive components, providing a potential therapeutic strategy for MASLD.

## Materials and methods

### Chemicals and reagents

Biochemical kits triglyceride (TG), total cholesterol (TC), low-density lipoprotein cholesterol (LDL-C), high-density lipoprotein cholesterol (HDL-C), aspartate transaminase (AST), and alanine aminotransferase (ALT), nonestesterified fatty acid (NEFA), superoxide dismutase (SOD), malondialdehyde (MDA), and glutathione (GSH) and commercial enzyme-linked immunosorbent assay (ELISA) kits apolipoprotein B (ApoB), very low density lipoprotein (VLDL), tumor necrosis factor-α (TNF-α), interleukin-1β (IL-1β), and interleukin-6 (IL-6) were purchased from Nanjing Jiancheng Bioengineering Institute (Nanjing, China). Insulin ELISA kit was purchased from CUSABIO (Wuhan, China). INSR, AKT, p-AKT, PPARγ, ACCα, and FASN antibodies were purchased from Immunoway (Plano, USA). PI3K p85, CYP7A1 and β-Actin antibodies were purchased from Affinity (Jiangsu, China). 2-methylbutyric acid was obtained from Solarbio (Beijing, China). Naringenin (NAR, purity of ≥ 95%) was purchased from sigma-Aldrich (St. Louis, MO, USA). Poly(ethylene glycol)-b-poly(d, l-lactide) (PEG-b-PLA) was purchased from Guangzhou Kelan Biotechnology Co. (Guangdong, China). 1,2-dioleoyl-3-trimethylammonium-propane chloride (DOTAP) was purchased from AVT Pharmaceutical Tech Co. (Shanghai, China). All other laboratory agents were used as analytical reagents.

### Animals and treatment

Male C57BL/6J mice (aged five weeks) were obtained from Southern Medical University (Guangzhou, China) (No. SCXK (Yue) 2021-0041) and housed in a specific pathogen-free (SPF) environment under a 12-h light/dark cycle with free access to water and standard chow. All animal experiments were approved by the Experimental Animal Ethics Review Committee of South China Agricultural University (approval number: 2023b059).

After one-week acclimatization period, mice were randomly divided into two groups: the normal chow (NC) group (*n* = 8), fed with a normal chow diet (NCD, Jiangsu Xietong Pharmaceutical Bio-engineering Co., Ltd., China); the HFD group (*n* = 32), fed with an high-fat diet (HFD, PD6001, 60% kcal fat, SYSE BIO) for 12 weeks to establish the MASLD model. HFD-fed mice were randomly divided into 4 groups (*n* = 8/group): model chow (MC) group, fed with a HFD with corn oil (4 mL/kg·d); the positive control group (PC), fed with a HFD with Vitamin E (150 mg/kg·d); NAR group NAR), fed a HFD with NAR (50 mg/kg·d); the NP-NAR group (NP-NAR), fed with a HFD with NP-NAR (50 mg/kg·d, based on NAR). NC group to be provided NCD with corn oil (4 mL/kg·d). All drugs and corn oil were administered to mice via oral gavage. The body weight and food intake were monitored weekly during the experimental. After 8 weeks, all mice were sacrificed. Blood samples were collected by removing the eyeballs of mice and centrifuged for serum extraction. Liver, epididymal fat, and perirenal fat tissues were then taken out and weighed. A portion of the liver tissue was transferred to formalin for assessment of morphological alterations, while the remaining liver samples were snap-frozen in liquid nitrogen and stored at -80 °C for subsequent analyses.

### Preparation and characterization of NP-NAR

As previously reported [22], NP-NAR was prepared by a double emulsion-solvent evaporation technique. Simply, PEG-b-PLA (168 mg), 0.3125 mg of DOTAP (2 mg) and NAR (16 mg) were dissolved with 3.6 mL mixed solvent of chloroform/ acetone (v/v: 5/1). The mixture was then mixed with ultrapure water at a ratio of 1:8 (v/v) and emulsified sonicated on an ice bath using probe sonication (Vibra-Cell VCX130, Sonics & Materials, Inc., USA). Finally, the residual organic solution was eliminated using a rotary evaporator (RV 10 digital V, IKA) at 37℃under vacuum. The size distribution and zeta potential of cationic nanoparticles loaded and unloaded with NAR were determined by dynamic light scattering (DLS) using a Malvern Zetasizer Nano ZSE (Malvern Instruments, UK) [23].

### Biochemical analysis

The levels of serum TG, TC, LDL-C, HDL-C, ALT, and AST were measured by colorimetry assays following the instructions of the supplier. Liver tissue was homogenized (1:9, w/v) in cold saline, centrifuged at 3000 rpm for 10 min, and the supernatant was taken. Then, the supernatant was used for measuring the hepatic levels of TG, SOD, MDA, GSH and NEFA with commercial kits. Furthermore, the hepatic Apo B, VLDL, TNF-α, IL-1β, and IL-6 were measured using ELISA kits.

### Glucose and insulin tolerance analyses

Oral glucose tolerance test (OGTT) and insulin tolerance test (ITT) were performed on the first day at 7 and 8 weeks, respectively. Mice were fasted overnight with free access to water and then orally administered 2 g/kg glucose or intraperitoneally injected with insulin (0.75 IU/kg). Blood glucose levels were measured at 0, 30, 60, 90, and 120 min using a glucometer (Yuyue, Danyang, China). Fasting serum insulin levels were determined using an ELISA kit. Homeostatic model assessment of insulin resistance (HOMA-IR) and homeostasis model assessment of β-cell function (HOMA-β) were calculated using following formulas:

1 $$\eqalign{{\rm{HOMA - IR = }}\, & {\rm{fasting }}\,{\rm{blood }}\,{\rm{glucose}}\,{\rm{ }}\left( {{\rm{mmol/L}}} \right)\, \cr& \times \,{\rm{fasting}}\,{\rm{ insulin}}\,{\rm{ }}\left( {{\rm{mIU/L}}} \right){\rm{/22}}{\rm{.5}} \cr} $$

2 $$\eqalign{{\rm{HOMA - \beta }}\,{\rm{ = }} & {\rm{20}}\, \cr& & & {\rm{ \times }}\,\,{{{\rm{fasting}}\,{\rm{insulin}}\,\left( {{\rm{mmol/L}}} \right)} \over {\left( {{\rm{fasting}}\,{\rm{blood}}\,{\rm{glucose}}\,\left( {{\rm{mmol/L}}} \right){\rm{ - 3}}{\rm{.5}}} \right)}} \cr} $$

### Histopathological evaluation

Freshly liver tissues were fixed in formalin, embedded in paraffin, sliced at 5 μm, and stained with hematoxylin and eosin (H&E) and Oil Red O. All images were visualized using an Olympus CX-31 Upright microscope (Olympus Corporation, Japan), and the MASLD activity score (NAS) criteria were utilized for the histologic scores [24].

### Transcriptomics analysis

RNA were extracted using TRIzol reagent, and the quality of RNA samples was assessed using the 2100 Bioanalyzer (Agilent, USA). Samples were then subjected to high-throughput RNA sequencing on an Illumina HiSeq 4000 instrument at Novogene (China). DESeq2 was used to screen differentially expressed genes (DEGs) based on a filter criteria of fold change (FC) ≥ 1 and qvalue < 0.05. Bioinformatics analysis included Venn analysis, DEGs screening, Gene Ontology (GO) enrichment analysis, Kyoto Encyclopedia of Genes and Genomes (KEGG) enrichment analysis, gene set enrichment analysis (GESA) analysis, and Protein − protein interaction (PPI) analysis.

### Quantitative real-time polymerase chain reaction (qRT-PCR)

Total RNA was acquired using an animal total RNA isolation kit (Foregene, China). Then, the mRNA was used to synthesize cDNA using cDNA synthesis kit (Applied Biological Materials, Canada). The mRNA levels were determined by the 2^−ΔΔCt^ equation and β-actin as the reference gene [25]. The primer sequences are displayed in STable S1 of the Supporting Information.

### Western blotting analysis

Total protein of liver tissues was extracted using RIPA lysis buffer and quantified using a BCA assay. The protein extracts were separated by 12% SDS-PAGE gels and transferred to Polyvinylidene Fluoride (PVDF) membranes. Subsequently, the membranes were blocked using skimmed milk for 2 h and then incubated overnight at 4 °C with primary antibodies (β-actin, PPAR γ, FASN, CYP7A1, ACCα, AKT, p-AKT, INSR, and PI3K p85). After washing thrice with Tris-buffered saline with 0.1% Tween 20 (TBST), the membranes incubated with secondary antibody diluted in TBAT at room temperature for 2 h. Protein bands were visluzalized using the ultra-sensitive enhanced chemiluminescent reagent (Biosharp) [26].

### SCFAs in fecal contents

The method for extracting and determining SCFAs was adjusted based on Shi [27]. Fecal samples (20 mg) and internal standard (10 µL, 2,2-dimethylbutyric acid) were mixed with HCl (400 µL, 1 M). After vortexing and three consecutive freeze-thaw cycles, the mixture was fvigorously extracted with 400 µL of diethyl ether. Following centrifugation (12000 rpm, 10 min, 4 °C), the supernatant was collected and 20 µL of bis(trimethylsilyl)trifluoroacetamide (BSTFA). After thorough mixing, the sample was allowed to stand at room temperature for 8 h prior to measurement. The sample was analyzed on Agilent 7890B-5977 A GC-MS (Agilent Technologies Co., Ltd., USA) equipped with a HP-5ms capillary GC column (30 m × 0.25 mm × 0.25 μm film thickness) (Agilent Technologies). 1 µL of the prepared sample was automatically injected into the inlet, maintained at 250 °C with a 10:1 split ratio. The carrier gas, high purity helium, was delivered at a flow rate of 1.0 ml/min. The programmed column temperature was as follows: the initial temperature of the column temperature box was 50 °C. the temperature of oven was increased to 150 °C at 15 °C /min, then 20 °C /min after reaching 150 °C, and finally at 5 °C/min until it reached 250 °C, when it was maintained for 5 min. The mass spectrometer employed an electron bombardment ion source (EI) and operated in full SIM mode with an electron energy of 70 eV. The quantification of SCFAs was carried out by using calibration curves of internal standards (2,2-dimethylbutyric acid) and calculating the amount in nanomoles of SCFA present per gram of cecal contents.

### Gut microbiota analysis

Fresh fecal samples were collected in the last 2 days to analyze gut microbiota, and extract total genomic DNA using the cetyltrimethylammonium bromide (CTAB) method. The PRC amplification of bacterial 16 S rRNA gene V4 region was performed with designated primers (F: GTGCCAGCMGCCGCGGTAA, R: GGACTACHVGGGTWTCTAAT), whose sequencing was carried out utilizing the Illumina MiSeq platform (Illumina, San Diego, USA) at the Technical Support Department of Nuohe Zhiyuan Biological Information Technology Co., Ltd. (Tianjin, China). Principal coordinate analysis (PCoA), and non-metric multidimensional scaling (NMDS) were performed and visualized on the basis of the R language platform. α-diversity indices, including Simpson index and Shannon diversity index were used to estimate microbial diversity within individual samples. The KEGG pathway of the gut microbiota was predicted using Fax4Fun.

### Statistical analysis

Data are presented as mean ± standard deviation (SD) for the indicated number of independently performed experiments. For normally distributed data, One-way ANOVA followed by the Tukey test using Graph Pad Prism 10 (GraphPad Software, CA, USA) was applied. Data with abnormal distribution in transcriptomics analysis and gut microbiota analysis were compared with the the Kruskal–Wallis test and Mann-Whitney U test abnormal distribution. Differences were regarded as statistically significant at *p*-value<0.05.

## Results

### Preparation and characterization of nanoparticles

The measurements of nanoparticles by dynamic light scattering indicated that NP-NAR with larger particle size than NP-Blank, but the polydispersity indices (PDI) of nanoparticles were not strong associated with the load material (STable S2). Due to the cationic nature of DOTAP makes the nanoparticles positively charged. Recent data show that nanoparticles less than 200 nm in size will be restricted from exiting normal vasculature but will still be able to enter the liver. Further, the nanoparticles carry a very slight positive charge can minimize nonspecific interactions and prevent nanoparticle loss to undesired locations [28].

### NP-NAR improved metabolic disturbance symptoms

**Fig. 1:**
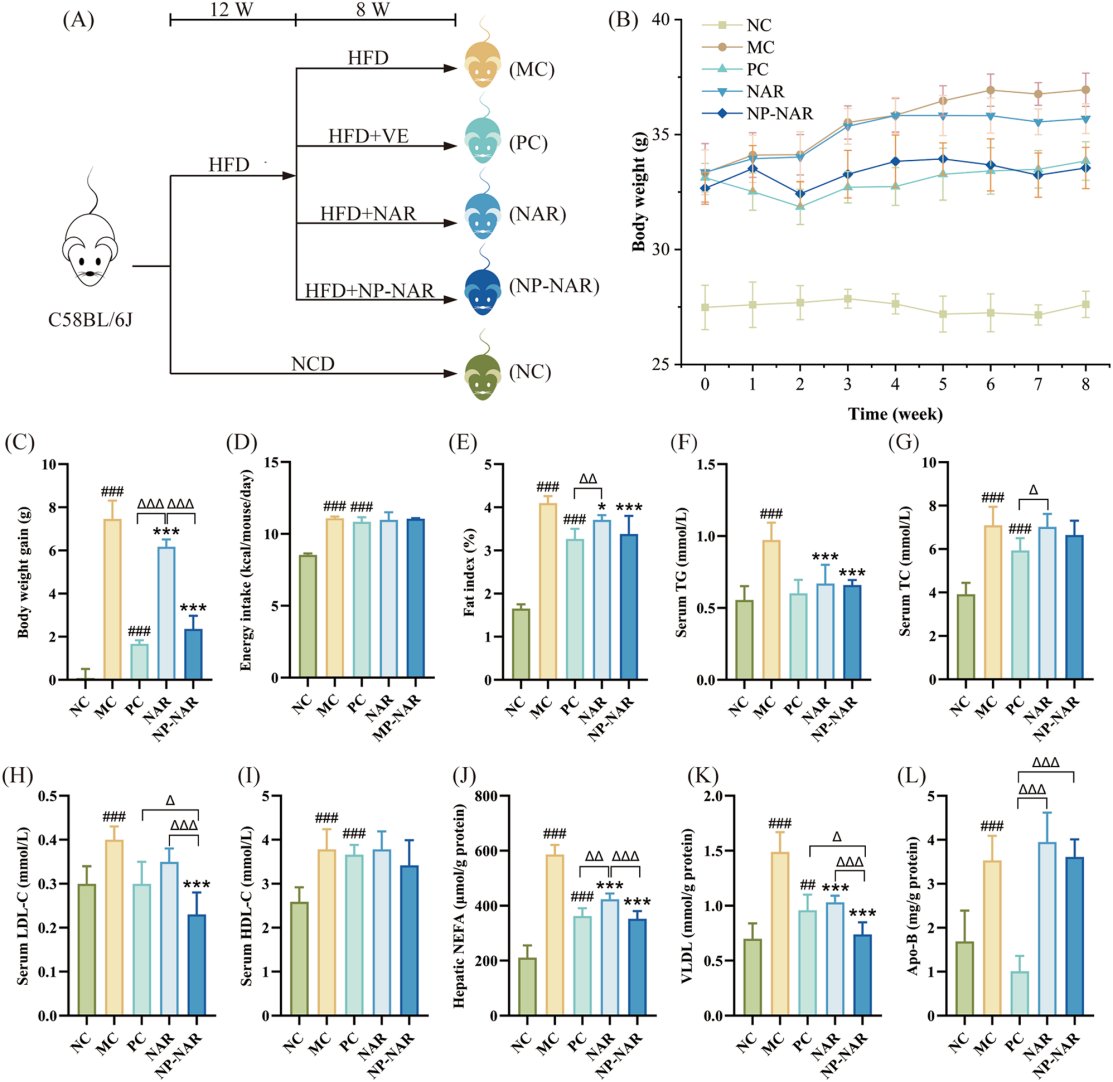
NP-NAR alleviated obesity and metabolic syndrome. (**A**) the simplified flow chart of MASLD mice model establishment and dietary interventions. (**B**) weight gain curves. (**C**) body weight gain. (**D**) energy intake. (**E**) fat index. Serum concentrations of TG (**F**), TC (**G**), LDL-C (**H**), and HDL-C (**I**). Liver concentrations of NEFA (**J**). VLDL (**K**), and Apo-B (L). Each value was expressed as the mean ± SD (*n* = 8). (#) *p* < 0.05, (##) *p* < 0.01, and (###) *p* < 0.001 compared to the NC group. (∗) *p* < 0.05, (∗∗) *p* < 0.01, and (∗∗∗) *p* < 0.001 in relation to the MC group. (Δ) *p* < 0.05, (ΔΔ) *p* < 0.01, and (ΔΔΔ) *p* < 0.001

In the 20-week modeling and intervention experiment, HFD-fed mice developed typical features of MASLD, including significant increases in body weight, fat accumulation, and dyslipidemias, all of which were effectively ameliorated by NP-NAR (Fig. 1). Both NAR and NP-NAR substantially inhibited body weight growth (*p* < 0.001, Fig. 1B-C) and fat accumulation (*p* < 0.05; *p* < 0.001, Fig. 1E) in MASLD mice, with NP-NAR showing a more pronounced effect in inhibiting weight gain (*p* < 0.001), and interestingly, they did not alter energy intake (Fig. 1D), indicating that NAR was independent of appetite suppression to control body weight. NP-NAR exhibited more effectively in controlling body weight and fat accumulation compared to NAR and approached the outcomes of the PC group. Dyslipidemia was a major risk factor for cardiovascular diseases and severe diseases in other organ systems, including MASLD and acute pancreatitis [29]. Compared with the MC group, both NAR and NP-NAR interventions showed a significant decline in TG content (*p* < 0.001, Fig. 1F), and a decreasing trend in TC and HDL-C (Fig. 1G, I). Notably, the LDL-C content of the NAR group was not significantly different from that of the MC group (*p* > 0.05), while a significant decrease in LDL-C content in the NP-NAR group and its effect was significantly better than that of the NAR and PC groups (*p* < 0.001; *p* < 0.05 Fig. 1H). Simple steatosis is directly caused by the conversion of hepatic NEFA to triglycerides and their accumulation [30]. HFD increased hepatic NEFA and VLDL, and Apo-B levels compared to the NC group (*p* < 0.001). Conversely, NAR and NP-NAR interventions significantly reduced NEFA and VLDL levels (*p* < 0.001), with an increasing trend in Apo-B levels (Fig. 1J-L), and NP-NAR acted significantly better than NAR in lowering hepatic NEFA and VLDL (*p* < 0.001).

### NP-NAR alleviates liver injury

**Fig. 2:**
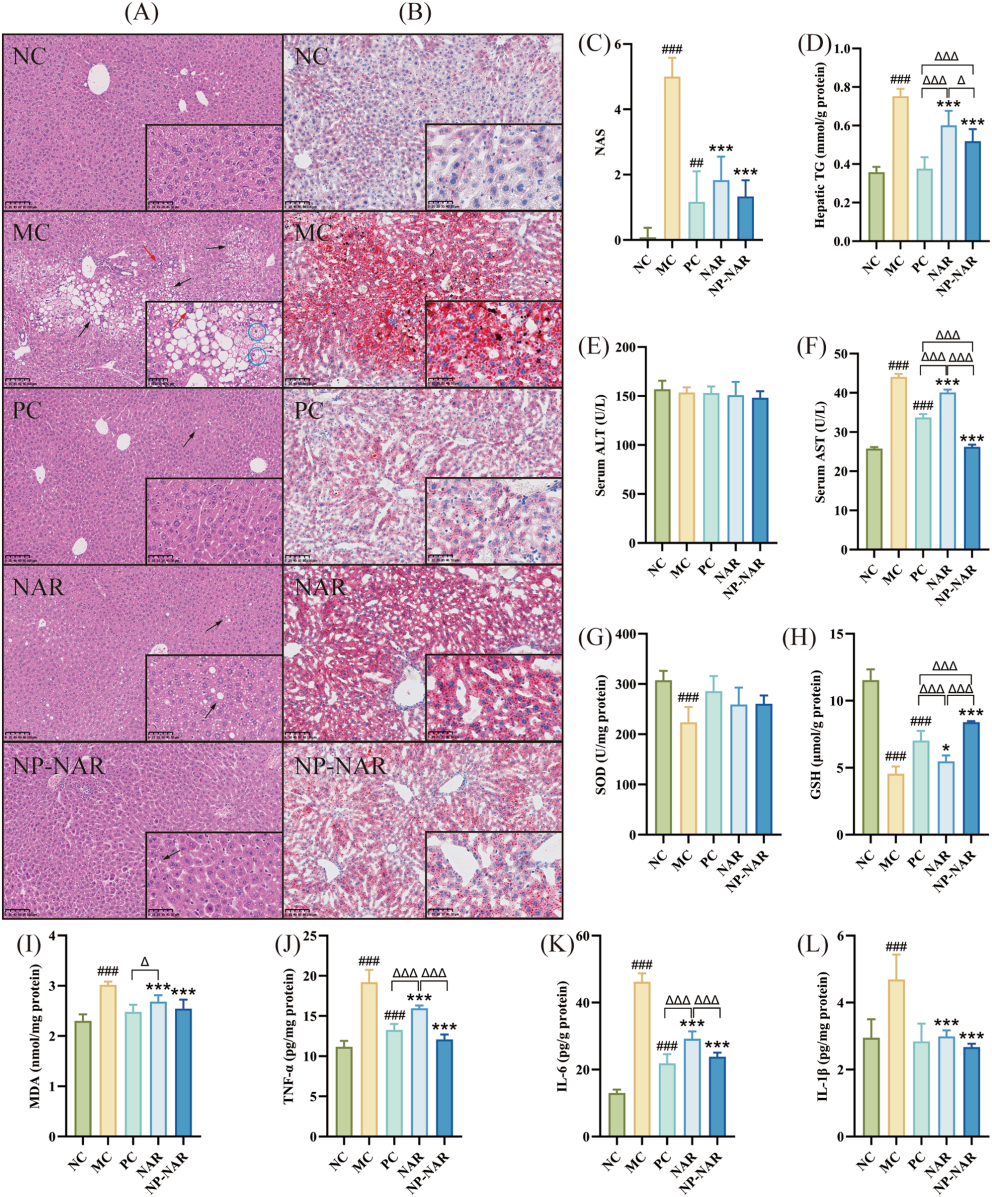
NP-NAR alleviates MASLD. (**A**-**B**) H&E and oil red O staining of liver tissue sections. (**C**) NAS. Liver concentrations of TG (**D**). Serum concentrations of ALT (**E**) and AST (**F**). Levels of SOD (**G**), GSH (**H**), and MDA (**I**) in the liver. Levels of TNF-α (**J**), IL-6 (**K**) and IL-1β (**L**) in the liver. The black and red arrows indicate hepatic steatosis and inflammation, respectively, and the blue circles indicate ballooning of hepatocytes. Each value was expressed as the mean ± SD (*n* = 8). (#) *p* < 0.05, (##) *p* < 0.01, and (###) *p* < 0.001 in relation to the NC group. (∗) *p* < 0.05, (∗∗) *p* < 0.01, and (∗∗∗) *p* < 0.001 compared to the MC group. (Δ) *p* < 0.05, (ΔΔ) *p* < 0.01, and (ΔΔΔ) *p* < 0.001

As shown in Fig. 2A, hepatic steatosis, inflammation, and hepatocyte ballooning were clearly observed in the MC group. All treatment groups showed good intervention compared to the MC group, accompanied by significantly lower NAS (*p* < 0.001, Fig. 2C), in which a small amount of steatosis was still observed in the NAR group, but tissue section results were more normalized in the NP-NAR and PC groups. Combined with the analysis of the results of Oil red staining and TG content in liver (Fig. 2B, D), the NP-NAR and NAR treatment significantly reduced lipid deposition in the livers of MASLD mice (*p* < 0.001), and NP-NAR was more effective than NAR (*p* < 0.05). The liver function indices revealed no significant difference in ALT levels across the groups (*p* > 0.05, Fig. 2D), while AST levels were significantly higher in the MC group (*p* < 0.001). Following supplementation with NAR and NP-NAR, there was a significant reduction in the levels of AST (*p* < 0.001, Fig. 2E), and NP-NAR was more effective than NAR and VE (*p* < 0.001).

### NP-NAR alleviates hepatic oxidative stress (OS) and inflammation

There was a strict pathophysiologic link between OS and MASLD [31]. As a critical antioxidant defenses against hepatic oxidative stress, antioxidant enzymes such as SOD and GSH are essential for normal liver function and health [32]. MDA, a product of lipid peroxidation, is an indicator of hepatic lipid peroxidation. In MC group exhibited lower SOD and GSH activities and higher MDA levels compared to the NC group (*p* < 0.001, Fig. 2F-G). Treatment with NAR and NP-NAR significantly increased GSH activity (*p* < 0.05; *p* < 0.001) and reduced hepatic lipid peroxidation (*p* < 0.001), with NP-NAR showing significantly higher GSH levels than the NAR and PC group (*p* < 0.001).

Next, We examined inflammatory factor levels in MASLD mice (Fig. 2I-K). Consistent with the results of H&E liver tissue staining, the MC group showed the highest levels of inflammatory factors (TNF-α, IL-6 and IL-1β) (*p* < 0.001). Both NAR and NP-NAR treatments significantly inhibited the release of these pro-inflammatory factors (*p* < 0.001), with the levels of TNF-α and IL-6 significantly lower than that of the NAR group (*p* < 0.001), and close to that of the PC group after NP-NAR intervention (*p* > 0.05).

### Transcriptomic analysis of the effect of NP-NAR on hepatic gene expression profiles

**Fig. 3:**
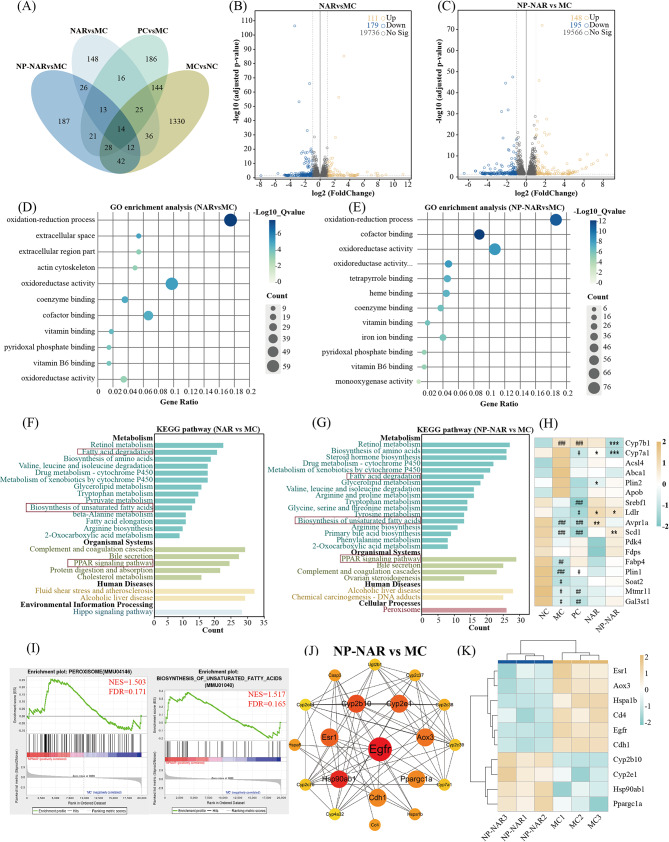
Effects of NP-NAR supplementation on hepatic transcriptome. (**A**) Venn diagram. (**B**-**C**) volcanic plot of DEGs in different groups. (**B**) NAR vs. MC groups. (**C**) NP-NAR vs. MC groups. DEGs were screened on *p*-value < 0.05 and | log_2_ (Fold Change)| > 1. (**D**-**E**) GO enrichment analysis in different groups. (**D**) NAR vs. MC groups. (**E**) NP-NAR vs. MC groups. (**F**-**G**) KEGG enrichment analysis in different groups. (**F**) NAR vs. MC groups. (**G**) NP-NAR vs. MC groups. (**H**) heat map analysis of lipid metabolism related genes across the groups. (**I**) GSEA enrichment analysis. (**J**) the PPI network with 20 hub genes in different groups. (**K**) heat map analysis of top 10 hug genes. (#) *p* < 0.05, (##) *p* < 0.01, and (###) *p* < 0.001 compared to the NC group. (∗) *p* < 0.05, (∗∗) *p* < 0.01, and (∗∗∗) *p* < 0.001 compared to the MC group

Transcriptomics was employed to analyze differential expression genes (DEGs) in hepatic cells under drug intervention, elucidating the mechanism by which NP-NAR alleviates MASLD. A Venn diagram showed the overall DEGs between the groups, revealing 87 and 96 genes could be reversed by NAR and NP-NAR, respectively (Fig. 3A). Compared to the MC group, NAR and NP-NAR up-regulated 111 and 148 genes and down-regulated 179 and 195 genes, respectively (Fig. 3B-C).

To explore DEGs enrichment pathways and functions, we completed GO, KEGG and GSEA enrichment analysis on the transcription results sequentially. GO analysis highlighted enrichment in oxidation-reduction processes, cofactor binding, and oxidoreductase activity for both NAR and NP-NAR groups compared to the MC group (Fig. 3D-E). KEGG enrichment analysis of the DEGs (Fig. 3F-G) showed that NAR and NP-NAR treatment enriched pathways associated with lipid metabolism, including fatty acid degradation, biosynthesis of unsaturated fatty acids and PPAR signaling pathway. Investigating the DEGs revealed that NP-NAR significantly increased the expressions of LDLR and SCD1 (*p* < 0.05, *p* < 0.01), and decreased the expressions of CYP7B1 and CYP7A1 (*p* < 0.001), as demonstrated in Fig. 3H. In addition, GSAE results revealed that NP-NAR positively promoted peroxisome and biosynthesis of unsaturated fatty acids (Fig. 3I). Peroxisome is the place for fatty acid oxidation and PPARs activity is intimately linked to MASLD, metabolic syndrome, and lipid metabolism [33]. Enhancement of the biosynthesis of unsaturated fatty acids pathway contributes to attenuation of liver injury, reduction of hepatic macrophage activation, neutrophil infiltration, and pro-inflammatory cytokine expression [34].

PPI network showed the top 20 hub genes in the NP-NAR vs. MC groups (Fig. 3J), The abbreviations and functions of top 10 hub genes were shown in STable S3. The top 10 hub genes’ expression variations between the MC and NP-NAR groups were displayed on the heatmap (Fig. 3K). Combined with Table S3, we found that NP-NAR treatment significantly up-regulated CYP2B10 and CYP2E1 (*p* < 0.05 or *p* < 0.01) and down-regulated EGFR, AOX3, CDH1, HSPA1B and CD4 (*p* < 0.05 or *p* < 0.01).

### Naringenin alleviates insulin resistance (IR)

**Fig. 4:**
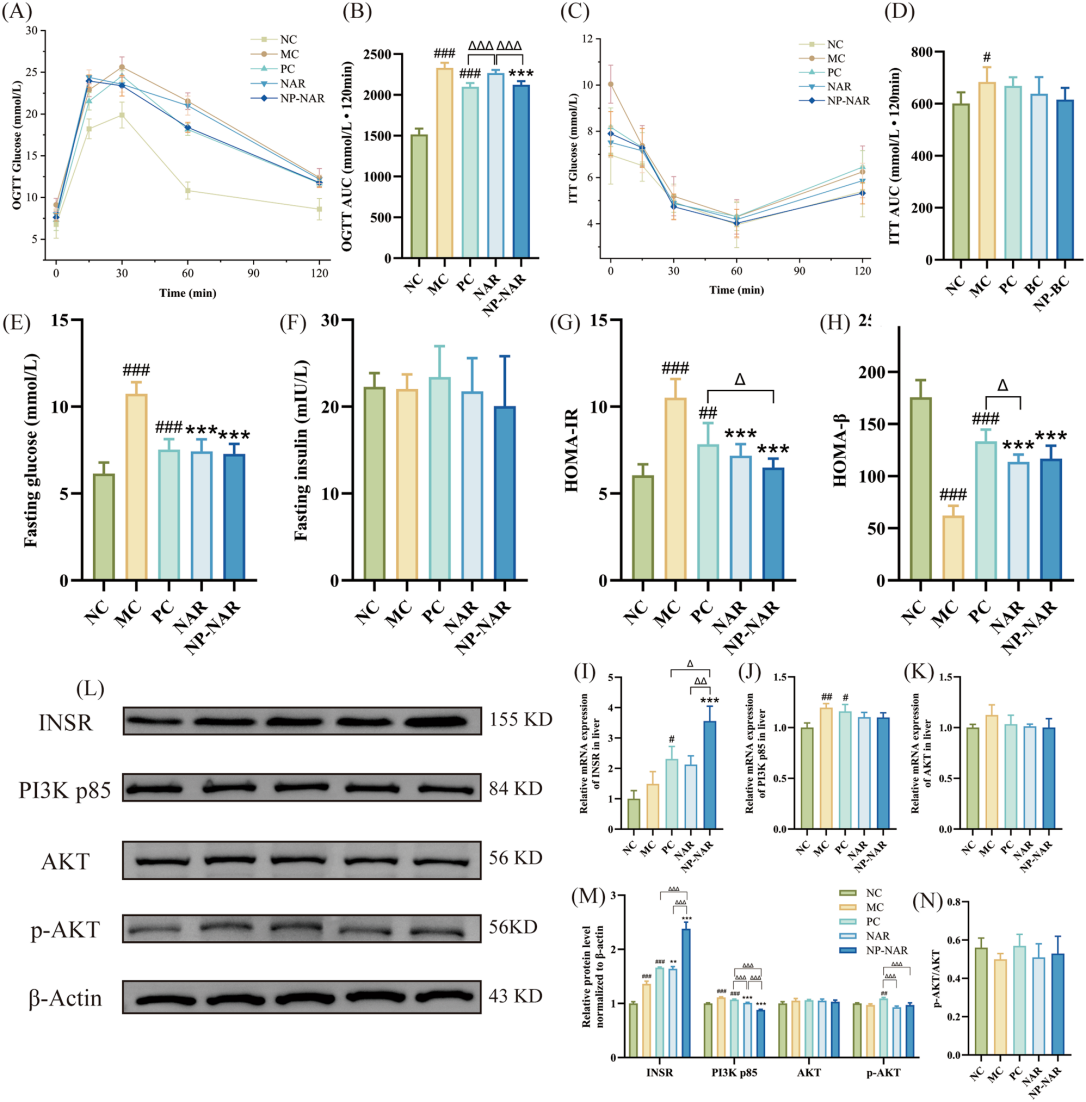
NP-NAR alleviates insulin resistance. (**A**) OGTT. (**B**) area under curve (AUC) analyses for OGTT. (**C**) ITT. (**D**) AUC analyses for ITT. (**E**) fasting glucose. (**F**) fasting insulin. (**G**) HOMA-IR. (**H**) HOMA-β. Each value was expressed as the mean ± SD (*n* = 8). Relative mRNA expression of INSR (**I**), PI3K p85 (**J**) and AKT (**K**) in liver tissue. (**L**-**M**) Western blot analysis of hepatic PI3K/AKT signaling pathways key proteins expression, β-Actin was used as the loading control. (**N**) p-AKT/AKT. Each value was expressed as the mean ± SD (*n* = 3). (#) *p* < 0.05, (##) *p* < 0.01, and (###) *p* < 0.001 in relation to the NC group. (∗) *p* < 0.05, (∗∗) *p* < 0.01, and (∗∗∗) *p* < 0.001 compared to the MC group. (Δ) *p* < 0.05, (ΔΔ) *p* < 0.01, and (ΔΔΔ) *p* < 0.001

IR is involved in the development and progression of MASLD from steatosis to NASH [35]. The OGTT curve showed that blood glucose increased sharply within 30 min after glucose gavage, and then began to decline slowly, in which the OGTT curve of the NC group was significantly lower than that of the other groups (Fig. 4A). The AUC analysis showed that the blood glucose concentration was significantly higher in the MC group than in the NC group. Compared to NAR group, NP-NAR significantly improved glucose tolerance in MASLD mice (*p* < 0.001), comparable to the PC group (*p* > 0.05, Fig. 4B). In fasted-state mice, blood glucose gradually decreased within 60 min after insulin injection, and then slowly increased, and the AUC results showed that HFD significantly increased insulin tolerance and decreased insulin sensitivity in mice (*p* < 0.05, Fig. 4C-D). MASLD mice (MC group) had the highest fasting blood glucose (10.67 ± 0.67 mmol/L), with some mice reaching 11 mmol/L, which was significantly higher than that in the other groups (*p* < 0.001, Fig. 4E). However, no significant difference in fasting insulin levels was observed among the groups (Fig. 4F). Furthermore, HOMA-IR and HOMA-β demonstrated that MASLD mice had lower index of pancreatic β-cell function and higher insulin resistance (*p* < 0.001, Fig. 4G-H). Nevertheless, NP-NAR and NAR were effectively regulated blood glucose levels and maintained glucose homeostasis in mice, thereby improving IR and pancreatic β-cell function (*p* < 0.001), and the NP-NAR intervention showed a better improvement trend.

To interpret the alleviating insulin resistance effect of NP-NAR, qPCR and western blot analyses of the insulin signaling pathway (PI3K/AKT signaling pathway) were performed. qPCR results showed that NP-NAR significantly increased the mRNA expression level of INSR (*p* < 0.001), while PI3K P85 and AKT mRNA expression showed a decreasing trend with no significance (*p* > 0.05). The immunoblotting results verified the activating effect on INSR and the inhibitory effect on PI3K p85 (*p* < 0.001), but there was no significant difference in p-AKT/AKT ratio between groups (*p* > 0.05), indicating that NP-NAR alleviated HFD-induced insulin resistance primarily by elevating INSR levels.

### NP-NAR regulates signaling pathways related to lipid metabolism

**Fig. 5:**
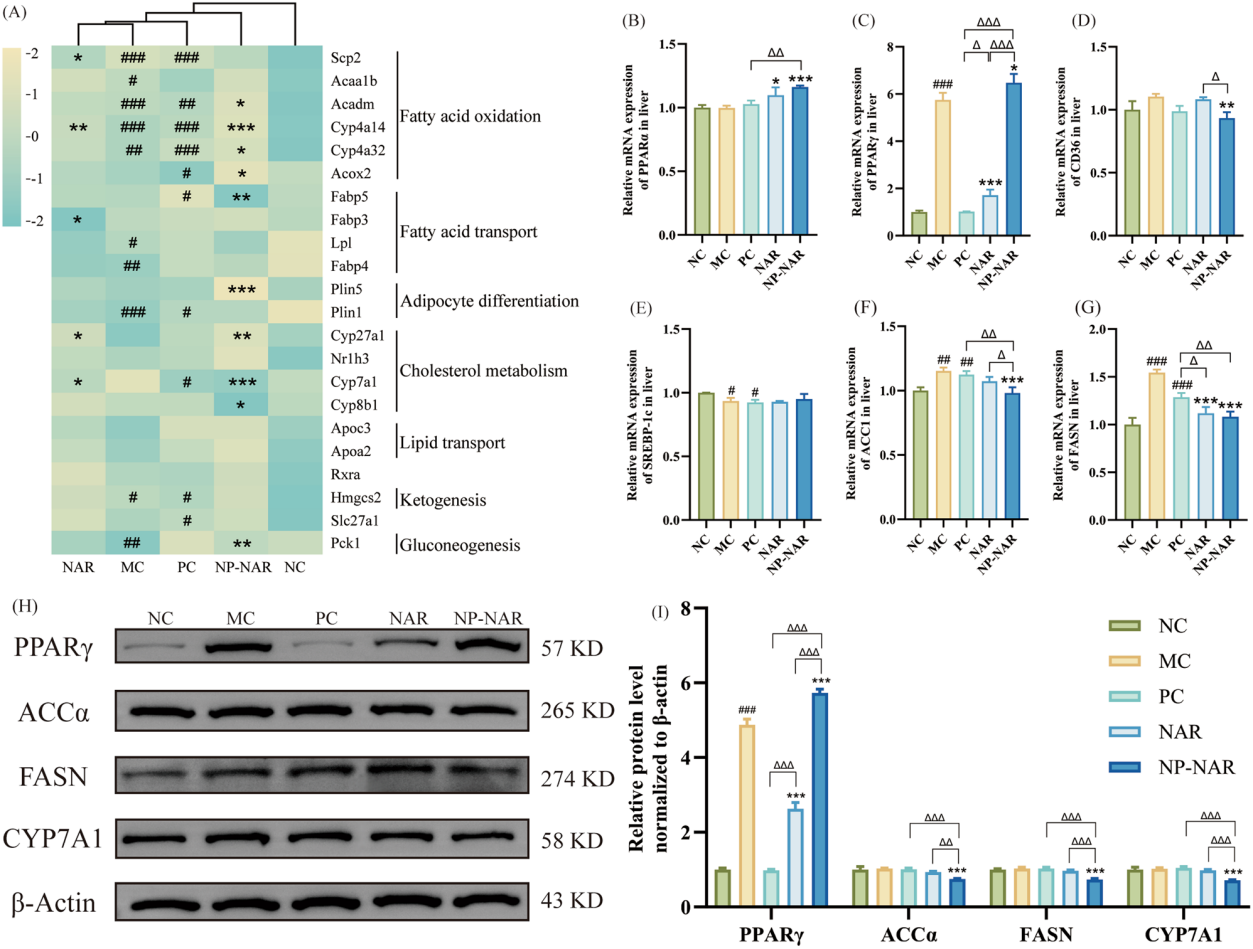
NP-NAR regulates signaling pathways related to lipid metabolism. (**A**) Heatmap of the relative expression levels of DEGs enriched in the PPAR signaling pathway. Relative mRNA expression of PPARα (**B**), PPARγ (**C**), CD36 (**D**), SREBP-1 C (**E**), ACC1 (**F**), and FASN (**G**) in liver tissue. (H-I) Western blot analysis of hepatic PPAR signaling pathways key proteins expression, β-Actin was used as the loading control. Each value was expressed as the mean ± SD (*n* = 3). (#) *p* < 0.05, (##) *p* < 0.01, and (###) *p* < 0.001 compared to the NC group. (∗) *p* < 0.05, (∗∗) *p* < 0.01, and (∗∗∗) *p* < 0.001 in relation to the MC group. (Δ) *p* < 0.05, (ΔΔ) *p* < 0.01, and (ΔΔΔ) *p* < 0.001

Based on the transcriptome sequencing results, we further explored the action mechanism of NP-NAR in regulating lipid metabolism in MASLD mice. DEGs in the NP-NAR group were enriched in the PPAR signaling pathway. Specifically, NP-NAR significantly regulated genes associated with fatty acid oxidation (ACADM, CYP4A14, CYP4A32, and ACOX2), fatty acid transport (FABP5), adipocyte differentiation (PLIN5), cholesterol metabolism (CYP27A1, CYP7A1, and CYP8B1) and gluconeogenesis (PCK1) (*p* < 0.05, *p* < 0.01, or *p* < 0.001, Fig. 5A). Consistent with transcriptome sequencing, qPCR analysis showed that NP-NAR significantly increased the mRNA expression levels of PPARα and PPARγ in the liver (*p* < 0.05 or *p* < 0.001) and decreased the expression levels of fatty acid uptake transporter CD36 mRNA in liver (*p* < 0.01, Fig. 5B-D). Compared to the NAR group, NP-NAR significantly up-regulated PPARγ and down-regulated CD36 genes expression (*p* < 0.001, *p* < 0.05). However, NP-NAR and NAR did not significantly affect SREBP-1 C mRNA expression (*p* > 0.05, Fig. 5E), although downstream genes of SREBP-1 C (ACC1 and FASN) showed decreased expression, especially in the NP-NAR group (*p* < 0.001, Fig. 5F-G). Western blot results also demonstrated that the NP-NAR intervention dramatically increased PPARγ protein content and significantly decreased ACCα, FASN, and CYP7A1 protein content (*p* < 0.001, Fig. H-I), while the NAR group significantly reduced PPARγ protein content (*p* < 0.001), and did not show significance in ACCα, FASN, and CYP7A1 protein content (*p* > 0.05).

### NP-NAR alleviates the structure of gut microbiota

**Fig. 6:**
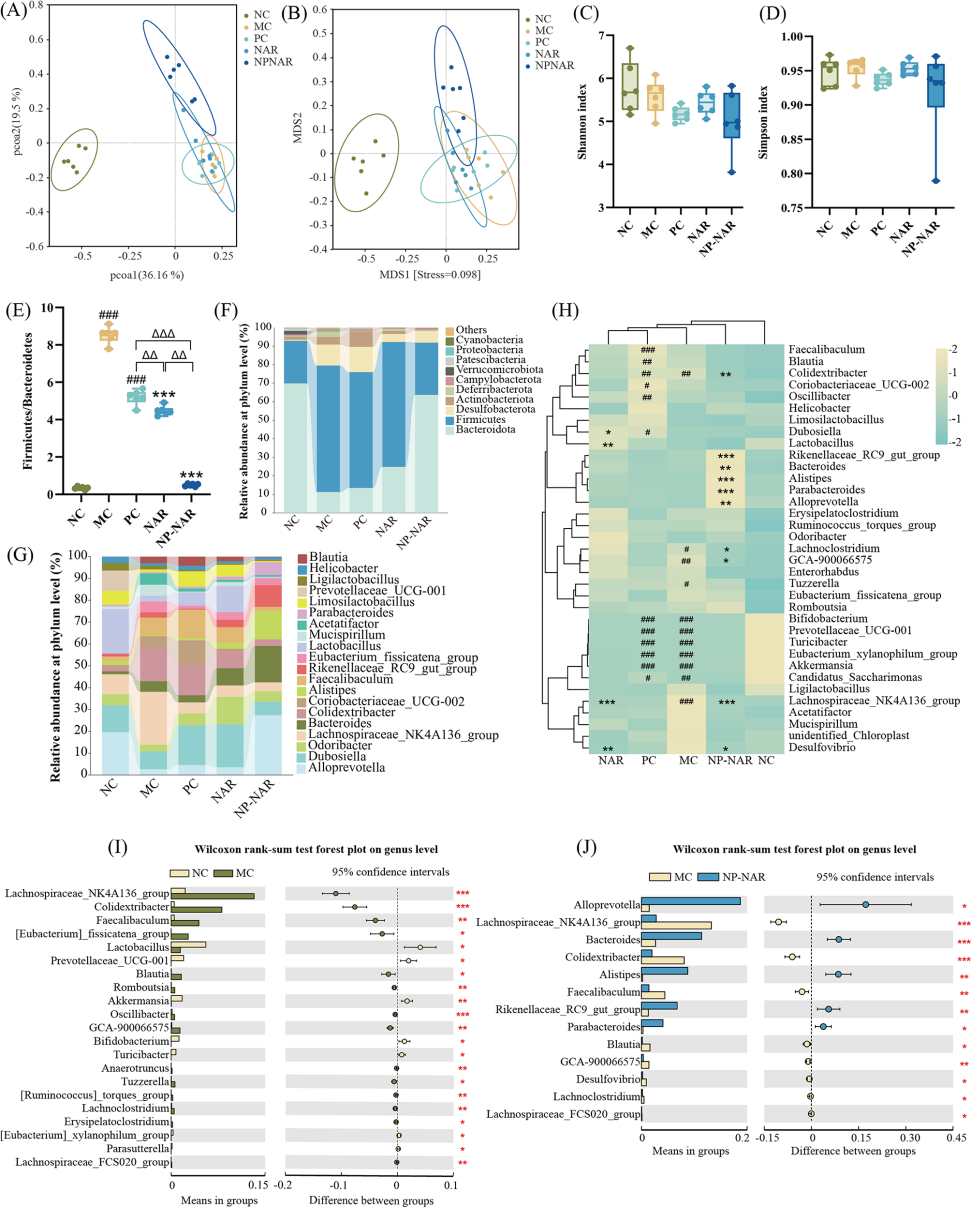
NP-NAR alleviates the structure of gut microbiota. (**A**) PCoA score plot based on weights. (**B**) NMDS score plot based on Bray − Curtis. (**C**) Shannon index. (**D**) Simpson index. (**E**) the F/B ratio. Relative abundances of intestinal microbes at the phylum level (**F**) and genus level (**G**). (**H**) Heatmap analysis of top 35 genus. Relative abundance of significantly different gut microbiota at the genus level between NC and MC groups (**I**) and NP-NAR and MC groups (**J**). Each value was expressed as the mean ± SD (*n* = 6). (#) *p* < 0.05, (##) *p* < 0.01, and (###) *p* < 0.001 compared to the NC group. (∗) *p* < 0.05, (∗∗) *p* < 0.01, and (∗∗∗) *p* < 0.001 in relation to the MC group. (Δ) *p* < 0.05, (ΔΔ) *p* < 0.01, and (ΔΔΔ) *p* < 0.001

MASLD patients often exhibit increased intestinal permeability and altered tight junctions, contributing to dysbiosis of the gut microbiota and the progression from hepatic steatosis to NASH [36]. Although gut microbial α-diversity assessment metrics (Shannon index and Simpson index) showed no significant difference in microbial diversity between groups (Fig. 6C-D), PCoA and NMDS demonstrated that HFD-fed significantly altered the gut microbiota profile, and supplementation with NP-NAR shifted the gut microbiota towards NC group (Fig. 6A-B).

At the phylum level, the MC group exhibited a decrease in *Bacteroidetes* abundance and an increase in *Firmicutes*, while were drastically reversed by treatment groups, especially the NP-NAR treatment (Fig. 6F). Accordingly, the NP-NAR treatment significantly reduced the ratio of *Firmicutes*/*Bacteroidetes* (F/B) in HFD-fed mice, which was significantly lower than in the NAR and PC groups (*p* < 0.01; *p* < 0.001 Fig. 6E) and close to that of the NC group (*p* < 0.001). The community barplot visualization displayed the top 20 gut microbiota species in each group at the genus level. It was observed that the MC group primarily raised the levels of *Firmicutes* by increasing the relative abundance of *Colidextribacter* and *Lachnospiraceae NK4A136 group*, while the NP-NAR group raised the levels of *Bacteroidetes* by increasing the relative abundance of *Alloprevotella*, *Bacteroides*, and *Alistipes* (Fig. 6G). A clustered heat map and the Wilcoxon rank sum test were used at the genus level to detect correlations and differentially described microorganisms between experimental groups (Fig. 6H-J). The structure of the intestinal flora was altered in all experimental groups under drug intervention. There were 21 differential microorganisms were detected in the MC and NC groups. Among them, *Lachnospiraceae NK4A136 group*, *Colidextribacter* and *Oscillibacter* showed the most significant differences in the MC and ND groups, respectively. In the MC versus NP-NAR group, the NP-NAR group strongly reduced the abundance of *Lachnospiraceae NK4A136 group*, *Colidextribacter*, *Facalibaculum*, *Blautia*, *GCA-900,066,575*,* Desulfovibrio*, *Lachnoclostridium*, and *Lachnospiraceae FCS020 group* and strongly increased the abundance of *Alloprevotella*, *Bacteroides*, *Alistipes*, *Rikenellaceae RC9 gur group*, and *Parabacteroides*, which contributes to the restoration of metabolism, amelioration of insulin resistance, modulation of cytokines, and the promotion of tight junctions to alleviate the pathologic changes in MASLD [37].

### NP-NAR regulated the concentrations of SCFAs

**Fig. 7:**
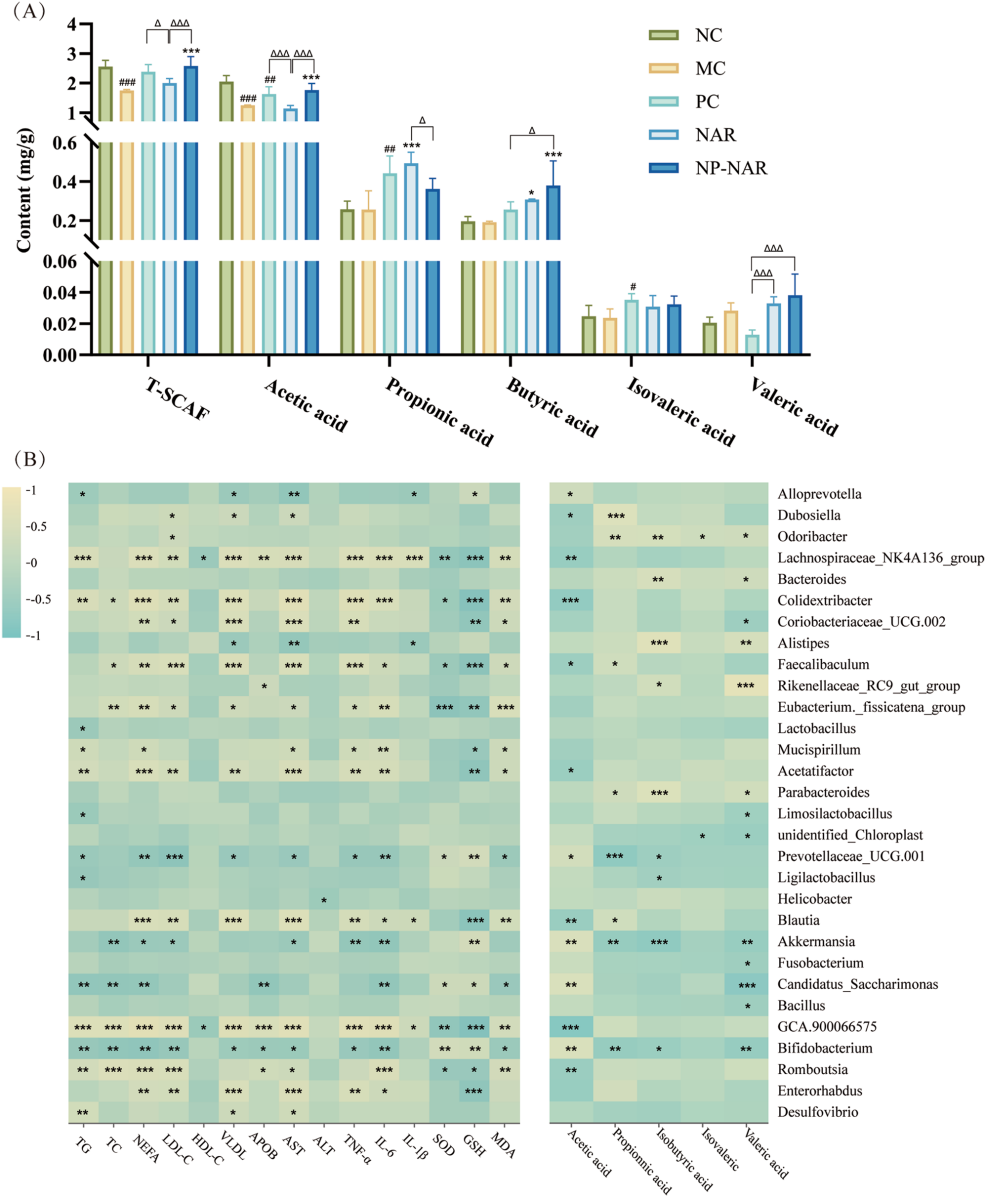
Correlation analysis of gut microbiota with MASLD-related metabolic parameters and SCFAs (*n* = 6). (**A**) SCFAs content. Each value was expressed as the mean ± SD. (#) *p* < 0.05, (##) *p* < 0.01, and (###) *p* < 0.001 in relation to the NC group. (∗) *p* < 0.05, (∗∗) *p* < 0.01, and (∗∗∗) *p* < 0.001 compared to the MC group. (Δ) *p* < 0.05, (ΔΔ) *p* < 0.01, and (ΔΔΔ) *p* < 0.001. (**B**) Spearman’s correlation heatmap analysis of the dominant gut microbiota (top 30 genera) with MASLD metabolic parameters. (∗) *p* < 0.05, (∗∗) *p* < 0.01, and (∗∗∗) *p* < 0.001

SCFAs are products of fermentation of dietary fiber by intestinal commensal bacteria [38]. As shown in Fig. 7A, the total content of SCFAs in the MC group was significantly lower than in the NC group, illustrated by the significant decrease in the content of acetic acid. Although NAR treatment significantly increased the concentrations of propionic and butyric acids (*p* < 0.001, *p* < 0.05), T-SCAT was not significantly different from the MC group (*p* > 0.05). In stark contrast, NP-NAR treatment significantly reversed the decrease in fecal SCFAs in MASLD mice (*p* < 0.001), by increasing the concentrations of acetic and butyric acids (*p* < 0.001). Thus, more than NAR, NP-NAR improved the intestinal microbiota and enhanced the production of SCFAs in the feces.

### Correlations between MASLD indices, SCFAs, and intestinal flora

Spearman correlation analysis was conducted to assess the correlation between gut microbiota with MASLD-related metabolic parameters and SCFAs. As indicated in Fig. 7B, further analysis of gut microbiota that primarily perform regulatory, such as *Lachnospiraceae NK4A136 group*, *Colidextribacter*, *Faecalibaculum*, *Eubacterium. fissicatena group*, *Acetatifactor*, *Blautia*, *GCA.900,066,575*, and *Romboutsia* were positively correlated with lipid metabolism (TG, TC, NEFA, LDL, HDL, VLDL, and APOB), liver function (AST and ALT), pro-inflammatory (TNF-α, IL-6, and IL-1β), antioxidant levels (SOD, GSH, and MDA), and SCFA production. In contrast, *Prevotellaceae CG.001*, *Akkermansia*, *Candidatus Saccharimonas*, and *Bifidobacterium* were negatively associated with lipid metabolism, liver function, pro-inflammatory, antioxidant levels, and SCFA production. This result confirmed that the content of SCFAs was negatively correlated with MASLD-related metabolic parameters.

### Modulatory effect of NP-NAR on HFD-induced KEGG pathways of gut microbiome

**Fig. 8:**
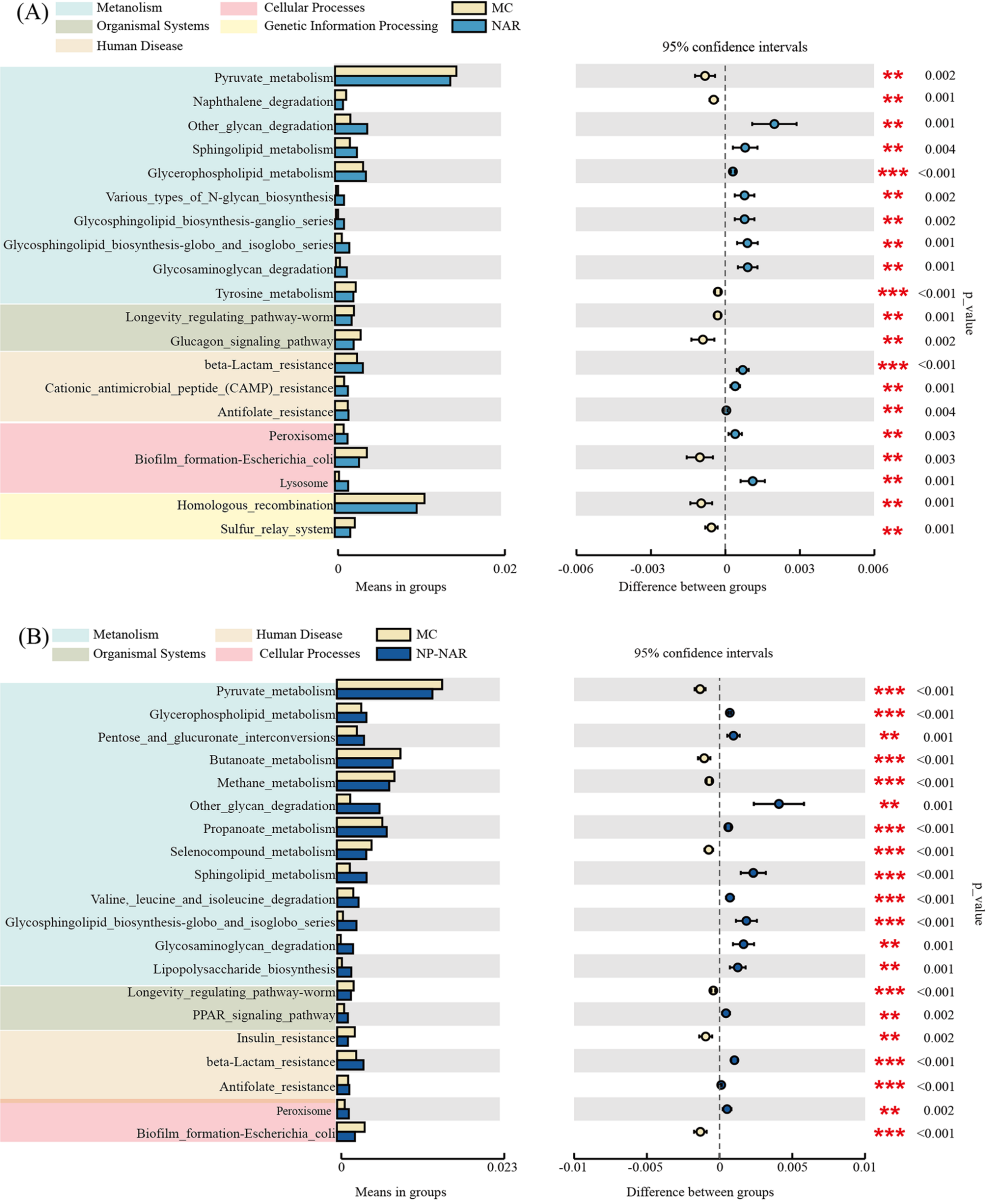
Top 20 KEGG pathways predicted to be significantly altered based on Tax4Fun analysis (*n* = 5). (**A**) pathway changed between the NAR and MC groups. (**B**) pathway changed between the NP-NAR and MC groups. (∗) *p* < 0.05, (∗∗) *p* < 0.01, and (∗∗∗) *p* < 0.001

Using Tax4Fun, the functional profile of the gut microbiota was predicted by analyzing changes in the microbial composition of each group. Glycerophospholipid metabolism, Tyrosine metabolism, and beta-Lactam resistance were the most significantly altered metabolism-related pathways after NAR treatment (*p* < 0.001, Fig. 8A). After treatment with NP-NAR, Changes in KEGG pathways were more pronounced, especially those related to energy and lipid metabolism. Shown in Fig. 8B, functional modules were enriched in NP-NAR group, including Valine, leucine, and isoleucine degradation, PPAR signaling pathway and Peroxisome, whereas the functional gene expression was significantly reduced in Pyruvate metabolism and Insulin resistance. The results of the Tax4Fun function prediction indicated that NP-NAR therapy helped to enhance energy and lipid metabolism, which in turn improved NAFLS symptoms.

## Discussion

The abnormal accumulation of TG in the liver is the first step towards MASLD, which triggers lipotoxicity, OS, and inflammatory response, and progresses to cirrhosis even liver cancer [39]. Unfortunately, despite advances in clinical drug development for MASLD, no pharmacotherapies for MASLD have been approved from the United States Food and Drug Administration [40]. Naringenin, a flavonoid compound known for its strong antioxidant and anti-inflammatory properties, has shown pharmacological effects in the treatment of obesity and associated metabolic disorders such as MASLD [41, 42]. However, naringenin’s hydrophobic nature, limits its therapeutic potential due to poor bioavailability. VE is recognized in current international guidelines as a pharmacological therapeutic option specific to MASLD [43]. The cationic nanoparticle system not only provides efficient encapsulation and protection but also extends the drug’s circulation time in the body and enhances its cellular uptake [12]. Here, we achieved efficient transport and targeted delivery of NAR with the help of cationic nanoparticle delivery system. VE served as a positive control drug, and we further assessed the therapeutic potential of NP-NAR in an HFD-induced MASLD mouse model.

The liver is a dynamic organ that plays critical roles in regulating systemic glucose and lipid metabolism [44]. Prolonged high-fat diet induces dysfunction of lipid metabolism and serologic features of hyperlipidemia (significant elevation of serum TG, TC, LDL-C levels and significant or non-significant reduction of HDL-C levels) [45]. In this study, we found that NP-NAR contributed more to the reduction of body weight and lipid accumulation than NAR, and that they did not do so by inhibiting energy intake, consistent with previous reports that demonstrated that naringenin intake did not affect appetite [46]. Furthermore, NAR encapsulated by nanoparticles is more helpful in reducing serum TG and LDL-C levels, and NEFA and TG content in the liver. Hepatic TG levels are maintained by the balance between lipid input (uptake of NEFA from circulation and de novo lipogenesis (DNL)) and output (secretion of VLDL and β-oxidation) [47]. ApoB is the key regulator of VLDL biosynthesis and secretion. HFD significantly increased hepatic levels of NEFA and APOB, contributing to the synthesis and secretion of VLDL and causing LDL-C accumulation throughout the VLDL-LDL delipidation cascade. Thus, HDL cholesterol-induced hepatic steatosis was not attributed to increased hepatic VLDL secretion impairment. NAR and NP-NRA had a tendency to increase Apo-B production, favoring the exclusion of excess TG in the liver.

Liver biopsy tissue examination remains the gold standard to confirm a diagnosis of MASLD [36]. Large areas of steatosis, multiple inflammatory foci, and ballooning were observed in pathologic sections of MASLD mice, whereas lipid accumulation in the liver was significantly prevented in treated groups, especially as no macrovesicular lipid droplets were seen in NP-NAR and VE treated mice. Previous studies have shown that naringenin can act as an Nrf activator to attenuate oxidative stress-induced hepatic injury in the liver, and also inhibits inflammation and prevents MASLD by down-regulating the NLRP3 / NF-κB pathway [7, 48]. The same results were obtained in our study, where NAR, especially nanoparticle-loaded NAR significantly increased the levels of endogenous antioxidant enzyme GSH and decreased the levels of lipid peroxides MDA, while significantly decreasing the levels of inflammatory factors (TNF-α, IL-6 and IL-1β), which led to the improvement of liver function in MASLD mice. Taken together, NP-NAR and NAR were effective in preventing hepatic lipid deposition, as well as excellent antioxidant and anti-inflammatory effects, with the effect of NP-NAR being more comparable to that of VE.

To further comprehend the improvement of NP-NAR on hepatic-associated metabolic processes, hepatic transcriptome analysis was performed. In the NP-NAR vs. MC, 343 DEGs were found (148 up-regulated and 194 down-regulated genes). These DEGs are predominantly enriched for GO terms (e.g., oxidation-reduction process, oxidoreductase activity, and monooxygenase activity) and KEGG pathways (e.g., fatty acid degradation, biosynthesis of unsaturated fatty acids, and PPAR signaling pathway) suggesting that NP-NAR may alleviate MASLD by regulating redox and lipid metabolism related genes. Further exploration of lipid metabolism gene expression in each group revealed that NP-NAR significantly decreased the expression of CYP7B1 and CYP7A1 and significantly increased the expression of LDLR and SCD1 compared to the MC group. CYP7B1 and CYP7A1 are associated with bile acid (BA) synthesis. In NASH mice, increased primary BA production by upregulating CYP7B1 and CYP7A1 make up for the low hepatic exposure to BA, but completely alters the balance between primary and secondary BA secreted by the liver [49]. In addition, the increase of LDL-C in the serum of mice in the MC group may also be related to the blocked expression of LDLR [50]. Notably, SCD1 was the key rate-limiting enzyme of lipid metabolism that synthesize lipid mediators, and absence of SCD1 significantly enhanced energy metabolism and resists weight gain induced by HFD [51]. GSEA enrichment analysis showed that NP-NAR significantly upregulated peroxisome and biosynthesis of unsaturated fatty acids pathways. Accumulation of long-chain fatty acids in hepatocytes triggers lipotoxicity and promotes the development of steatosis [52]. The peroxisome is an important site for β-oxidation of long-chain fatty acids, and a shortage of peroxisomes leads to dysregulated fatty acid oxidation and lipotoxicity [53]. In addition, unsaturated fatty acids help reduce inflammation in the body, which has a favorable impact on health [54]. The functional properties of NP-NAR and MC hub DEGs are mainly related to lipid metabolism and apoptosis. The combined results of various transcriptome analyses showed that NP-NAR ameliorated MASLD by regulating genes related to redox and lipid metabolism, and ameliorated lipotoxicity and hepatic TG accumulation mainly by promoting fatty acid β-oxidation.

Continued exposure to elevated glucose and lipids leads to β-cell dysfunction and even β-cell death, and the resulting decrease in insulin output increases glucolipotoxicity triggering a harmful vicious cycle [55]. In vitro and in vivo studies have shown that citrus flavonoids may serve as promising phytochemicals for targeting diabetes and related complications [56]. As expected, NP-NAR treatment significantly reduced fasting blood glucose values, IR, and repaired pancreatic β-cells. Ahmed et al. [57] stated that naringenin reduced IR, which may be mediated through enhancing INSR, GLUT4 and adiponectin expression. In this study, the level of INSR mRNA and protein expression in the liver of NP-NAR treated mice was significantly higher than that of the MC group. Decreased INSR levels ultimately led to cell dysfunction and insulin resistance, whereas NP-NAR’s elevated INSR levels in the liver improved HFD-induced IR.

The PPAR signaling pathway is involved in lipid transport, synthesis and oxidation, and is an important pathway in the regulation of lipid metabolism. Our study revealed that NP-NAR treatment significantly increased the mRNA expression levels of PPARα and PPARγ compared to the MC group. PPARα activates several enzymatic pathways, such as fatty acid transport, fatty acid oxidation, ketogenesis, and cholesterol metabolism, as indicated in the results of heat map analysis of the PPAR signaling pathway. PPARγ controls uptake of fatty acids and its activation helps ameliorate inflammation and oxidative stress [58]. The fatty acid receptor CD36 not only facilitates long-chain fatty acid uptake but also acts as a key regulator for SREBP-1 C-mediated de novo lipogenesis (DNL) in liver. Overexpression of CD36 contributes to the progression of MASLD [59, 60]. In our study, NP-NAR drastically reduced the level of liver CD36 mRNA, as well as DNL major enzymes content (e.g., ACCα and FASN). Changes in BA composition are closely associated with MASLD, synthesized from cholesterol in hepatocytes through two major pathways, the classical and alternative pathways. Zheng et al. [61] found that astragalus polysaccharide attenuated HFD-induced MASLD by inhibiting BA synthesis of key factors of the classical pathway (e.g., CYP7A1 and CYP8B1) by facilitating the alternative pathway. Our findings indicated that NP-NAR treatment inhibited the classical pathway, but whether it promoted the alternative pathway still needs to be further explored. Similar results were observed in the present study with NP-NAR treatment. Based on previous reports and our observations, it could be deduced that NP-NAR acted on multiple pathways of lipid metabolism: (1) activation of the PPAR signaling pathway to promote lipid oxidation; (2) inhibition of lipid uptake and lipid synthesis by blocking CD36 expression to reduce the levels of ACCα and FASN proteins in the liver; (3) inhibition of the classical pathway of BA synthesis to affect cholesterol metabolism.

Naringenin is primarily degraded in the colon into smaller, absorbable phenolics, which may exert beneficial effects on the intestinal microbiota [62–64]. HFD-induced metabolic disorders are usually accompanied by dysregulation of the composition and structure of the gut microbiota, such as an elevated *Firmicutes*/*Bacteroidetes* (F/B) ratio [26]. In this experiment, we observed that the treatment groups significantly reduced the HFD-induced increase in F/B ratios, with the most pronounced effect of NP-NAR, which was close to that of normal mice. Specifically, NP-NAR increased the abundance of *Alloprevotella*, *Bacteroude*, *Alistipes*, *Rikenellaceae RC9 gut group* and *Parabacteroides*, where *Alloprevotella*, *Bacteroude*, *Rikenellaceae RC9* gut group belong to SCFAs-producing bacterium [65, 66]. The oral administration of NP-NAR for 8 weeks significantly increased the levels of SCFAs. Previous studies have shown that Alistipes has a protective effect in diseases such as colitis and various liver and cardiovascular fibrosis diseases [67]. *Parabacteroides* imbalances are associated with a variety of diseases, including obesity and the development of metabolic dysfunction-associated steatotic liver disease (MASLD) [68]. In the present study, NP-NAR facilitated the growth of *Alloprevotella* and *Alistipes*, which exhibited negative correlations with VLDL, AST and TG, and IL-1β. In addition, we found that colonies with significant downregulation of NP-NAR (e.g., *Colidextribacter* and *Faecalibaculum*) were mostly positively correlated with indicators of lipid accumulation, liver function, oxidative stress and inflammation. Tax4Fun was used to predict the functional characteristics of gut microbiota based on changes in microbial composition between groups. Both Tax4Fun and KEGG enrichment analyses highlighted the PPAR signaling pathway, a key pathway in lipid metabolism, suggesting that NP-NAR can modulate intestinal flora to intervene in hepatic lipid metabolism pathways.

Present results presented here have clearly demonstrated that NP-NAR regulated hepatic lipid metabolism and related target proteins by modulating gut microbiota, while gut microbiota and NP-NAR worked together to alleviate NAFLD and obesity. However, this experiment still has some limitations and lacks a controlled study of cationic nanoparticles without NAR was added on NAFLD. Although the component of cationic nanoparticles are clinically validated or under clinical investigations, ensuring its safety profile for in vivo use, it is not possible to rule out the effect of the nanoparticles themselves on NAFLD in an absolute sense, and we will further refine our exploration of this element in the future.

## Conclusions

In summary, NP-NAR represents a significant advancement as a safe and efficient nanocarrier system. NP-NAR shows potential in ameliorating lipid deposition, metabolic dysfunction, oxidative stress, and inflammation in MASLD mice by reprogramming lipid metabolism and modulating gut flora and host metabolism. Therefore, this study suggests that the beneficial effects of NP-NAR are comparable to those of VE and it can be considered as a promising candidate for alleviating MASLD.

## Electronic supplementary material

Below is the link to the electronic supplementary material.

Supplementary Material 1

## Data Availability

The raw sequencing data generated in this study have been deposited in the NCBI Sequence Read Archive under the accession numbers PRJNA1151960.
