# Supplementary material for: Naringenin cationic lipid-modified nanoparticles mitigate MASLD progression by modulating lipid homeostasis and gut microbiota
Source: J Nanobiotechnology. 2025 Mar 4;23:168. doi: 10.1186/s12951-025-03228-x (PMC11881431; doi:10.1186/s12951-025-03228-x)
Supplement: Supplementary file 1 — Supplementary Material 1 [file 12951_2025_3228_MOESM1_ESM.docx]

**Supplement information for**

**Naringenin Cationic Lipid-Modified Nanoparticles Mitigate MASLD Progression by Modulating Lipid Homeostasis and Gut Microbiota**

**Lu Dong^a^, Wenyong Lou^a^, Congfei Xu^b*^ , Juan Wang^a*^**

^a^ School of Food Science and Engineering, South China University of Technology, Guangzhou 510641, China.

^b^ School of Biomedical Sciences and Engineering, South China University of Technology, Guangzhou International Campus, Guangzhou 511442, China.

* Corresponding author at: School of Biomedical and engineering, South China University of Technology, Guangzhou International Campus, Guangzhou, Guangdong Province, China. E-mail: xucf@scut.edu.cn (C. Xu)

School of Food Science and Engineering, South China University of Technology, Guangzhou, Guangdong Province, China. E-mail: wangjuan@scut.edu.cn (J. Wang)

**STable 1.** Primers Used for qRT-PCR Analysis

| primer | forward (5'-3') | reverse (3'-5') |
| --- | --- | --- |
| mSREBP-1c | GGATCGCAGTCTGAGGAGGAG | CCAGGAGCCGACAGGAAGG |
| mPPARγ | CCAAGAATACCAAAGTGCGATC | TCACAAGCATGAACTCCATAGT |
| mPPARα | ACGATGCTGTCCTCCTTGATGAAC | GATGTCACAGAACGGCTTCCTCAG |
| mACC1 | GCTCGTGTGTGGAAGTGGATGTG | TGGTGTAACTGCTGCCGTCATAAG |
| mCD36 | GCAGGTCTATCTACGCTGTGTTCG | TGTCTGGATTCTGGAGGGGTGATG |
| mFASN | TGCCCGAGTCAGAGAACCTACAG | TGCCCGAGTCAGAGAACCTACAG |
| mINSR | TCCGCCGCTCCTATGCTCTG | GAGTTGCCTCAGGTTCTGGTTGTC |
| mPI3K P85 | GGAATGTCGGGAGCAGCAACC | TCTACCACTACGGAGCAGGCATAG |
| mAKT | GCCACGCTACTTCCTCCTCAAG | GACTCTCGCTGATCCACATCCTG |

**STable 2.** Particle size analysis, zeta potential and polydispersity index (PDI) of cationic nanoparticles

|  | Size/nm | PDI | Zeta/mV |
| --- | --- | --- | --- |
| NP-Blank | 152.83±3.46 | 0.40±0.05 | 21.93±1.59 |
| NP-NAR | 168.11±0.51 | 0.36±0.02 | 20.46±0.18 |

**STable 3.** Functional Roles of 10 Hub Genes

| Hub gene | Pathway | GO | NP-NARvsMC |
| --- | --- | --- | --- |
| EGFR | MAPK signaling pathway and PI3K-AKT signaling pathway | trandferase activity and response to stimulus | ↓** |
| HSP90AB1 | PI3K-Akt signaling pathway and IL-17 signaling pathway | negative regulation of apoptotic process and regulation of cell cycle | - |
| CYP2B10 | steroid hormone biosynthesis and arachidonic acid metabolism | cellular ketone metabolic process and steroid metabolic process | ↑** |
| ESR1 | endocrine resistance and pathways in cancer | auxin-activated signaling pathway and cytokinin-activated signaling pathway | - |
| CYP2E1 | linoleic acid metabolism and arachidonic acid metabolism | long-chain fatty acid metabolic process and triglyceride metabolic process | ↑* |
| AOX3 | tyrosine metabolism and tryptophan metabolism | xenobiotic metabolic process | ↓* |
| CDH1 | hippo signaling pathway and gastric cancer | enzyme regulator activity and protein metabolic process | ↓** |
| PPARGC1A | AMPK signaling pathway and insulin signaling pathway | positive regulation of fatty acid oxidation and negative regulation of protein phosphorylation | - |
| HSPA1B | MAPK signaling pathway and antigen processing and presentation | negative regulation of apoptotic process and positive regulation of proteasomal ubiquitin-dependent protein catabolic process | ↓* |
| CD4 | T cell receptor signaling pathway and primary immunodeficiency | adaptive immune response and positive regulation of T cell activation | ↓** |
